# Comprehensive Assessment of Multidrug-Resistant and Extraintestinal Pathogenic *Escherichia coli* in Wastewater Treatment Plant Effluents

**DOI:** 10.3390/microorganisms12061119

**Published:** 2024-05-31

**Authors:** Ji-Hyun Park, Kyung-Seon Bae, Jihyun Kang, Jeong-Ki Yoon, Soo-Hyung Lee

**Affiliations:** 1 Han River Environment Research Center, National Institute of Environment Research, Yangpyeong-gun, Incheon 12585, Gyeonggi-do, Republic of Korea; 2 Division of Water Supply and Sewerage Research, National Institute of Environment Research, Yangpyeong-gun, Incheon 22689, Gyeonggi-do, Republic of Korea; bae1207@korea.kr (K.-S.B.); jhkang2@korea.kr (J.K.); jkyun@korea.kr (J.-K.Y.); lshnier@korea.kr (S.-H.L.)

**Keywords:** antibiotic resistance gene, virulence gene, multilocus sequence types, biofilm, high-risk clones

## Abstract

Multidrug-resistant (MDR) *Escherichia coli* poses a significant threat to public health, contributing to elevated rates of morbidity, mortality, and economic burden. This study focused on investigating the antibiotic resistance profiles, resistance and virulence gene distributions, biofilm formation capabilities, and sequence types of *E. coli* strains resistant to six or more antibiotic classes. Among 918 strains isolated from 33 wastewater treatment plants (WWTPs), 53.6% (492/918) demonstrated resistance, 32.5% (298/918) were MDR, and over 8% (74/918) were resistant to six or more antibiotic classes, exhibiting complete resistance to ampicillin and over 90% to sulfisoxazole, nalidixic acid, and tetracycline. Key resistance genes identified included *sul2*, *bla*_TEM_, *tetA*, *strA*, *strB*, and *fimH* as the predominant virulence genes linked to cell adhesion but limited biofilm formation; 69% showed no biofilm formation, and approximately 3% were strong producers. Antibiotic residue analysis detected ciprofloxacin, sulfamethoxazole, and trimethoprim in all 33 WWTPs. Multilocus sequence typing analysis identified 29 genotypes, predominantly ST131, ST1193, ST38, and ST69, as high-risk clones of extraintestinal pathogenic *E. coli*. This study provided a comprehensive analysis of antibiotic resistance in MDR *E. coli* isolated from WWTPs, emphasizing the need for ongoing surveillance and research to effectively manage antibiotic resistance.

## 1. Introduction

Multidrug-resistant (MDR) bacteria pose a significant threat to public health [1]. MDR is characteristically defined as resistance to three or more classes of antimicrobial agents [2]. MDR bacteria are the predominant etiological determinants responsible for therapeutic failure in the context of infectious diseases, thereby engendering increased duration and magnitude of morbidity, mortality rates, and economic burden on healthcare systems [3]. MDR bacteria are commonly linked to infections acquired in healthcare settings, and some species have increasingly become the common cause of community-wide infection [1].

Wastewater treatment plants (WWTPs) play a crucial role as reservoirs and origins of antibiotic-resistant bacteria, as well as antibiotic resistance genes that govern their resistance [4]. The infiltration of antibiotics and their metabolites into wastewater primarily stems from the overuse of antibiotics, improper disposal methods, and the excretion of pharmaceuticals by humans and animals [5]. Additionally, pharmaceutical compounds and resistant bacteria can infiltrate wastewater treatment systems through the discharge of hospital, industrial, and residential wastewater, which is eventually released into the environment. Research on WWTPs as crucial sites for the spread of antibiotic and multidrug resistance has been ongoing [6].

*Escherichia coli* is a prominent pathogenic micro-organism that causes various infections, including increasingly severe cases linked to antimicrobial-resistant strains that contribute to rising morbidity and mortality rates [7]. *E. coli* plays a crucial role in the dissemination of antibiotic resistance and serves as a valuable indicator of transmission pathways because of its widespread distribution, cohabitation in ecological niches similar to those of other enteric pathogens, and potential transmission through shared routes [8]. Although *E. coli* is intrinsically susceptible to most clinically relevant antimicrobial agents, it possesses a remarkable capacity to accumulate resistance genes through horizontal gene transfer [9]; this allows it to acquire diverse genetic elements, such as plasmids, integrons, and transposons, thereby gaining a broad spectrum of antibiotic resistance genes and developing multidrug resistance [10]. Currently, the increasing prevalence of multidrug resistance in *E. coli* is a global concern affecting human and veterinary medicine [9]. MDR *E. coli* strains may experience selective pressure in human, animal, and environmental settings, leading to the emergence of high-risk MDR *E. coli* lineages. These high-risk clones exhibit enduring fitness and widespread dissemination, associated with significant variability in resistance and virulence genes [10].

Extraintestinal pathogenic *E. coli* (ExPEC) lineages are the primary causative agents of a majority of extraintestinal infections in humans worldwide, leading to substantial direct healthcare expenses and societal burdens. Although ExPEC strains encompass a multitude of lineages, only a specific subset is responsible for most infections [11]. Prior research has demonstrated that strains affiliated with phylogroups A and B1 are typically commensal, whereas those associated with phylogroups B2, D, and E exhibit characteristics consistent with those of extraintestinal pathogenic strains [12,13]. The predominant approach for discerning clonal complexes or lineages associated with ExPEC is multilocus sequence typing (MLST) [11]. A multitude of sequence types (ST)s and clonal complexes (CC) have been characterized based on the genetic markers of *E. coli*. The most commonly documented lineages include ST131, ST69, ST10, ST405, ST38, ST95, ST648, ST73, and ST1193, which were identified in infections associated with healthcare and community settings [10]. Thus far, investigations on MDR ExPEC strains have predominantly focused on isolates derived from human samples, while there is a scarcity of comprehensive studies simultaneously assessing MDR ExPEC using culture-based methods with effluents from WWTPs.

The objective of this study was to evaluate the antibiotic resistance of strains isolated from WWTP effluent, particularly those that exhibited resistance to six or more antibiotic subclasses. Additionally, we aimed to ascertain the prevalence of antibiotic resistance and virulence genes within these strains, as well as evaluate their biofilm-forming ability. Furthermore, we aimed to identify the predominant sequence type through MLST analysis.

## 2. Materials and Methods

### 2.1. Isolation and Identification of E. coli

In 2021, effluent samples were collected from 33 WWTPs in Korea. To isolate pure strains of *E. coli* from these samples, a 100-μL aliquot of the wastewater effluent was streaked onto CHROMagar™ orientation agar (CHROMagar, Paris, France) plates using a disposable bacterial loop. Following an incubation period of 24 h at 35 °C, we observed pink colonies and subsequently transferred them onto MUG Nutrient Agar (Difco Laboratories, Sparks, MD, USA) plates for species identification. After 24 h of incubation, fluorescent colonies were selected using ultraviolet illumination. To confirm that the isolated strains were *E. coli*, we performed either 16S rRNA sequencing or matrix-assisted laser desorption ionization time-of-flight mass spectrometry (MALDI-TOF/MS) [14].

### 2.2. Phenotyping of Antibiotic Resistance

The phenotypic profiles of the *E. coli* isolates were assessed using the microdilution method, which is a common technique for testing antimicrobial susceptibility. Bacterial suspensions adjusted to a 0.5 McFarland standard were introduced into a 96-well microtitration plate (KRNV5F, Daejeon, South Korea), which is commercially available for use in the livestock industry and contains 16 antimicrobial agents. Following a 1-day incubation at 35 °C, the minimum inhibitory concentration (MIC) was determined either through visual inspection or using an automated reader. This reader greatly simplifies reading microdilution tests and ensures accurate recording of results by effectively identifying microbial growth within the wells. The MIC endpoints were determined based on the predefined breakpoints for the 16 antimicrobial agents in the panel according to the guidelines established by the Clinical Laboratory Standard Institute [15] and the European Committee on Antimicrobial Susceptibility Testing [16]. The antibiotic resistance rate was calculated as a percentage, representing the number of strains exhibiting values above the MIC breakpoint for each antibiotic divided by the total number of strains.

### 2.3. Detection of Antibiotic Resistance Genes and Virulence Genes

A DNeasy Plant Mini Kit (Qiagen Inc., Valencia, CA, USA) was used to extract genomic bacterial DNA. Genes responsible for resistance to tetracycline (*tetA*, *tetB*) [17], streptomycin (*strA*, *strB*) [18], aminoglycoside (*aadA*) [19], sulfonamides (*sul1, sul2, and sul3*) [20], β-lactamase (CTX-M-groups [21], *bla*_TEM_, *bla*_OXA-1_, *bla*_OXA-2_ [22], *bla*_SHV_, and *bla*_CMY-2_ [23]), and quinolone (*qnrA*, *qnrB*, *qnrC*, *qnrD*, *qnrS*, *and qnrVC*) [24] were investigated using polymerase chain reaction (PCR) (Table S1).

To examine the distribution of the virulence genes, 14 genes were selected based on their relevance to various virulence mechanisms, including bacterial adhesion (*fimH*, *papC*, *papGII*, *eaeA*, and *afa/draBC*); iron acquisition (*iroN*, *fyuA*); toxin and hemolysin production (*stx1*, *stx2*, and *east1*); invasion (*ibeA*, *ipaH*); serum resistance (*traT*); and capsule synthesis (*kpsMTII*) [25,26,27] (Table S1).

### 2.4. Determination of Phylogroups

Phylogenetic characterization of all *E. coli* isolates (designated A, B1, B2, C, D, E, and F) was performed using quadruplex PCR with four specific phylogenetic markers, *chuA*, *yjaA*, *TspE4.C2*, and *arpA*. This approach closely followed the procedure established by Clermont et al. [28]. Additionally, secondary PCR was performed to differentiate between groups E and C, targeting the *arpA* and *trpA* regions, respectively. Furthermore, groups B2 and F were subdivided into a newly identified group designated G using a triplex PCR assay that encompassed the *trpA*, *cfaB*, and *ybgD* markers, in accordance with the methodology proposed by Clermont et al. [29].

### 2.5. Multilocus Sequence Typing Analysis

Seven housekeeping genes, *adk*, *fumC*, *gyrB*, *icd*, *mdh*, *purA*, and *recA*, were amplified using the recommended primers [30]. The products were purified using a QIAquick PCR Purification Kit (Qiagen Inc., Hilden, Germany) and sequenced by the Macrogen Sequencing Service (Macrogen, Seoul, Republic of Korea). Allele numbers for the seven gene fragments of each isolate were determined via comparative analysis with the corresponding alleles accessible at on https://pubmlst.org/ (accessed on 4 October 2023). The allelic numbers and their corresponding genotype designations were allocated by the curator on the MLST website.

### 2.6. Biofilm Analysis

Biofilm formation assays were performed as described previously [31,32,33], with some modifications. Bacterial strains were cultured in Muller Hinton Agar and standardized to a density of 0.5 McFarland units in 0.85% NaCl medium. A 10-µL aliquot of each suspension was diluted 1:20 in 190 µL of Luria-Bertani (LB) medium in 96-well plates. Following incubation at 37 °C for 24 and 48 h, the plates underwent triple washing with 0.85% NaCl medium, and the remaining adherent bacteria were fixed with 200 µL of methanol per well. After air drying, the wells were stained with 0.1% crystal violet for 20 min. Subsequently, the wells were rinsed with distilled water and air dried. The crystal violet dye bound to the attached cells was resuspended in 200 µL of ethanol, and optical density (OD) was measured at 550 nm.

Biofilm formation was categorized as negative, weak, moderate, or strong. The cut-off value (ODc) was defined as the mean OD value above three standard deviations of the negative control. All experiments were performed in triplicate, and the results were averaged. The OD values measured at 595 nm for the negative controls served as the ODc, following a previously described method [31,32,33], with slight modifications. The biofilm-forming ability was classified as follows: OD < ODc, nonbiofilm producers; ODc < OD < 2 × ODc, weak producers; 2 × ODc < OD < 4 × ODc, moderate producers; and OD > 4 × ODc, strong producers.

### 2.7. Determination of Antimicrobial Residue

The 16 antimicrobial residues in the wastewater samples were determined as follows: Initially, each sample (500 mL) was pretreated via filtration through a 0.2-μm PVDF filter. Subsequently, 900 µL of the filtered sample was transferred into amber autosampler vials. To this, 100 µL of a 1% acetic acid solution, 40 mg/mL of ethylene diaminetetra-acetic acid disodium salt dihydrate (Na_2_EDTA), and 10 µL of 10 ng/mL isotopically labeled standards were added. The resulting pretreated sample (200 μL) was then subjected to high-performance liquid chromatography coupled with tandem mass spectrometry [34]. The presence of residual antibiotics and their average concentration in WWTPs from 33 sampled facilities have been delineated in the results.

### 2.8. Statistical Analysis

To examine the relationship between phenotypes and genotypes, we conducted a chi-square test. This statistical test was chosen to determine if there is a significant association between the categorical variables under study. All statistical analyses were performed with a significance level set at *p* < 0.05. Specifically, the chi-square test results were considered statistically significant if *p* < 0.001.

## 3. Results

### 3.1. Antibiotic Resistance Profiles and Multidrug-Resistant Phenotypes

We performed antibiotic susceptibility testing on 918 strains of *E. coli* isolated from 33 wastewater effluent samples in 2021, targeting 16 antibiotics representing 13 antibiotic subclasses. The antibiotic resistance rates of all isolated strains were as follows: ampicillin, 34.5% (317/918); nalidixic acid, 32.6% (299/918); tetracycline, 28.9% (265/918); sulfisoxazole, 25.9% (238/918); streptomycin, 23.4% (215/918); and trimethoprim/sulfamethoxazole, 20.7% (190/918). Among the 918 strains, 53.6% (492/918) exhibited resistance, with MDR strains accounting for 32.5% (298/918) of that subset. In addition, over 8% (74/918) of the strains exhibited resistance to six or more antibiotic classes.

**Figure 1:**
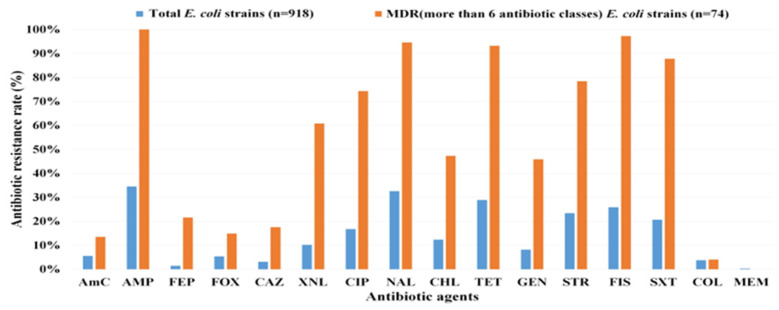
Antibiotic resistance rates. Blue bar: proportion of resistant strains among 918 *E. coli* strains from wastewater effluents. Orange bar: proportion of resistant strains among 74 strains resistant to six or more antibiotic classes from the same 918 *E. coli* isolates. AmC, Amoxicillin/clavulanic acid; AMP, Ampicillin; FEP, Cefepime; Fox, Cefoxitin; CAZ, Ceftazidime; XNL, Ceftiofur; CIP, Ciprofloxacin; NAL, Nalidixic acid; CHL, Chloramphenicol; TET, Tetracycline; GEN, Gentamicin; STR, Streptomycin; FIS, Sulfisoxazole; SXT, Trimethoprim/Sulfamethoxazole; COL, Colistin; MEM, Meropenem.

Of the strains subjected to resistance assessment, 74 strains that exhibited resistance to six or more antibiotic subclasses were used to comprehensively assess the characteristics of *E. coli* exhibiting strong MDR. The antibiotic resistance patterns of the 74 *E. coli* strains to 16 antibiotics are shown in Figure 1. The highest resistance was conferred against ampicillin (100% of the strains, 74/74), followed by sulfisoxazole (97.3%, 72/74), nalidixic acid (94.6%, 70/74), and tetracycline (93.2%, 69/74). The least prevalent resistance among all strains was for meropenem (0%), which is a broad-spectrum carbapenem, followed by colistin (4.1%, 3/74) (Table S2).

### 3.2. Antibiotic Resistance Genes and Virulence Genes in MDR E. coli

**Figure 2:**
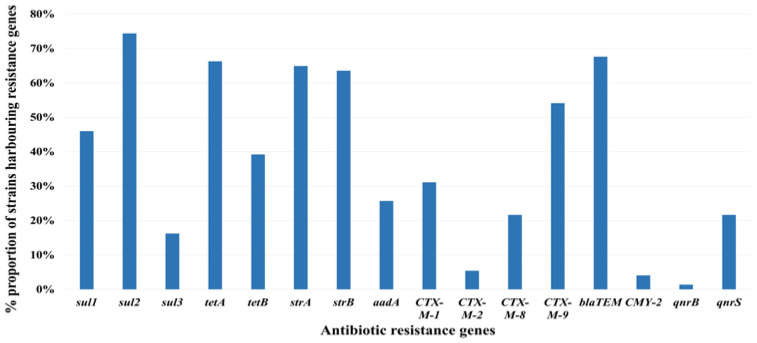
Antibiotic resistance gene profile in multidrug-resistant *E. coli*.

Among the antibiotic resistance genes examined, the following genes had the most prominent prevalence rates: *sul2* (74.3%, 55/74), *bla*_TEM_ (67.6%, 50/74), *tetA* (66.2%, 49/74), *strA* (64.9%, 48/74), and *strB* (63.5%, 47/74). The prevalence of the remaining antibiotic resistance genes is shown in Figure 2. We observed distinct prevalence patterns across various antibiotic classes using antibiotic resistance gene profiling. In the sulfonamide group, *sul2* exhibited the highest prevalence, followed by *sul1* and *sul3*. In the tetracycline class, *tetA* exhibited greater prevalence than *tetB*. For streptomycin-related resistance genes, *strA* and *strB* were detected in over 60% of the cases. In the beta-lactamase group, specifically within the CTX-M family, the CTX-M-9 group was dominant, accounting for 54.1% (40/74) of the prevalence, followed by the CTX-M-1 (31.1%, 23/74), CTX-M-8 (21.6%, 16/74), and CTX-M-2 (5.4%, 4/74) groups. Notably, the presence of CTX-M-25 was not confirmed. Moreover, *bla*_TEM_ exhibited the highest prevalence rate (67.6%), whereas *bla*_CMY-2_ was detected at a frequency of 4.1%. However, *bla*_OXA-1_, *bla*_OXA-2_, and *bla*_SHV_ were not detected. We also examined the antibiotic resistance genes associated with quinolones, namely *qnrA*, *qnrB*, *qnrC*, *qnrD*, *qnrS*, and *qnrVC*. Intriguingly, only *qnrS* (21.6%, 16/74) and *qnrB* (1.4%, 1/74) were detected, suggesting a relatively limited presence of quinolone resistance genes.

The chi-squared test revealed a statistically significant association between cefoxitin and ceftazidime resistance and *bla*_CMY-2_; ceftiofur resistance and *bla*_TEM_; and nalidixic acid resistance and *qnrS* (*p* < 0.001). Additionally, streptomycin resistance was associated with *tetA*, *tetB*, *strA*, *strB*, and *bla*_TEM_ (*p* < 0.001).

We screened 14 virulence genes, and the results are presented in Table 1. *fimH* had the highest prevalence (97.3%), followed by *fyuA*, *traT* (both at 59.5%), and *kpsMTII* (43.2%). Notably, *ipaH*, *stx1*, and *stx2* were not detected. In the context of functional categorization, analysis of the screened virulence genes revealed that the genes associated with bacterial adhesion, *fimH*, and iron acquisition, *fyuA*, were prevalent in most strains. Additionally, *traT*, associated with serum resistance, was detected in over 50% of the strains, whereas *east1* was associated with toxin and hemolysin production.

### 3.3. Phylogenetic Characterization and MLST Analysis

**Figure 3:**
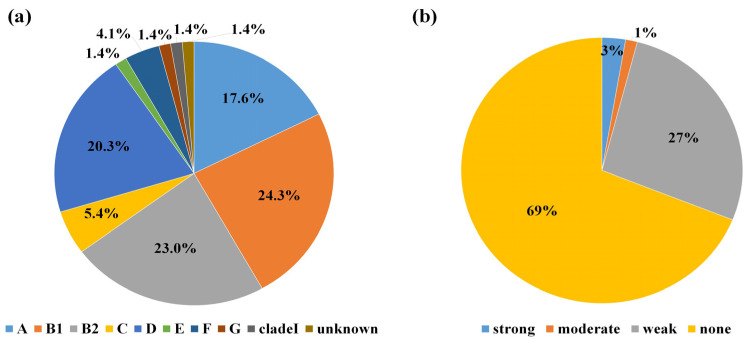
(**a**) Distribution of phylogenetic groups classified by Clermont et al. [28,29]: Proportion of strains belonging to each phylogroup (A, B1, B2, C, D, E, F, G, clade I, unknown) among the multidrug-resistant 74 *E. coli* strains; (**b**) Distribution of biofilm-forming abilities: Proportion of strains classified by biofilm formation (Strong, moderate, weak, none) among the multidrug-resistant 74 *E. coli* strains.

Phylogenetic analysis results for the MDR *E. coli* strains isolated from WWTP effluents are presented in Figure 3a. Overall, most *E. coli* strains were assigned to nine phylogroups: B1 (24.3%, 18/74), B2 (23%, 17/74), D (20.3%, 15/74), A (17.6%, 13/74), C (5.4%, 4/74), F (4.1%, 3/74), E (1.4%, 1/74), G (1.4%, 1/74), and clade I (1.4%, 1/74). The remaining (1.4%, 1/74) *E. coli* strains were categorized as unclassified.

**Figure 4:**
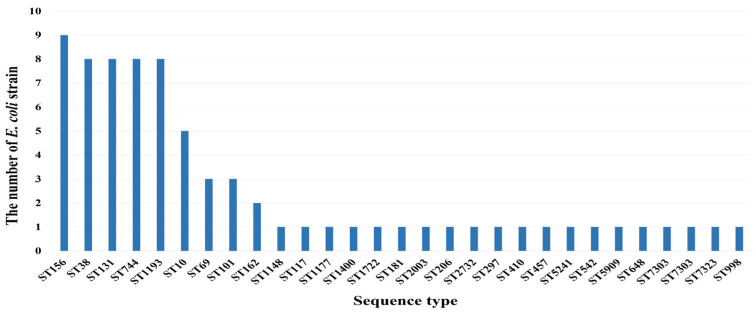
MLST genotypes of 74 multidrug-resistant *E. coli* strains.

Among the 74 MDR *E. coli* strains from WWTP effluents, a total of 29 MLST genotypes were identified; ST156 (12.2%), ST38 (10.8%), ST131 (10.8%), ST744 (10.8%), and ST1193 (10.8%) were the predominant genotypes. ST10 accounted for 6.8%, whereas ST69 and ST101 each represented 4.1%. The results of the MLST analysis are shown in Figure 4.

We assessed antibiotic resistance and the distribution of resistance and virulence genes in recognized extraintestinal pathogenic high-risk international clones, namely, ST131, ST1193, ST69, and ST38. The results are summarized in Table 2. Within phylogroup B2, except for one strain, all strains belonged to either ST131 or ST1193. ST131 exhibited a 100% prevalence of *bla*_TEM_ and *tetA*, with the majority of strains carrying the CTX-M-1 or CTX-M-9 genes. Additionally, all ST131 strains displayed a 100% presence of crucial virulence genes, including *fimH*, *fyuA*, and *traT*. ST1193 possessed *sul2*, *strA*, *strB*, *fimH*, *fyuA*, and *kpsMII*, each with a prevalence of 100%. ST69 and ST38 predominantly comprised phylogroup D. ST69 exhibited 100% prevalence of *fimH*, *fyuA*, and *traT* but lacked *bla*_TEM_. ST38 possessed the CTX-M-8, *fimH*, *fyuA*, and *kspMII* genes at 100% prevalence.

### 3.4. Assessment of Biofilm Formation Capability

Assessment of biofilm-forming proficiency demonstrated that only 31% of the examined samples were capable of forming biofilms. Within this subset, 27%, 1%, and 3% possessed a weak, moderate, and strong ability to form biofilms, respectively (Figure 3b).

### 3.5. Residual Antibiotic Measurement

We investigated the residual concentrations of 18 antibiotics during the dry and wet seasons, focusing on effluents from 33 WWTPs. The results are presented in Table 3. Seven antibiotics (ciprofloxacin, sulfamethoxazole, trimethoprim, ceftazidime, tetracycline, cefepime, and meropenem) were detected in the dry-season samples, whereas six antibiotics were detected in the wet-season samples, except for meropenem. Ciprofloxacin, sulfamethoxazole, and trimethoprim were detected at all 33 WWTP effluent sampling points.

## 4. Discussion

In this study, we investigated the antibiotic resistance rates of 918 *E. coli* strains isolated from WWTP effluent. We focused on the strains exhibiting resistance to six or more antibiotic classes. To our knowledge, this study is the first comprehensive analysis of antibiotic resistance in MDR *E. coli* isolated from WWTPs using culture-based methods, encompassing analyses of antibiotic resistance genes, virulence genes, biofilm formation ability, and MLST analysis for *E. coli* typing. Overall, *E. coli* isolates from the WWTPs exhibited antibiotic resistance in the following order: ampicillin, nalidixic acid, tetracycline, sulfisoxazole, and streptomycin. In the case of MDR strains, the major antibiotics with high resistance rates were nearly identical, albeit with different orders. MDR strains demonstrated increased resistance to most antibiotics. Moreover, our investigation revealed concurrent resistance, mirroring earlier research outcomes [35], wherein every chloramphenicol-resistant strain exhibited concomitant resistance to tetracycline and ampicillin. Notably, in contrast to the typical pattern observed for most antibiotics, colistin and meropenem did not manifest an inclination toward increased resistance rates in MDR strains. This suggests a potentially independent mechanism of colistin- and meropenem-induced resistance compared with that of other antibiotic classes. Recent studies have highlighted the significant resistance rates of *E. coli* strains to various antibiotics, underscoring an urgent concern regarding antibiotic resistance in WWTPs. In South Africa, *E. coli* exhibited a substantial resistance rate of 92.2% to sulfamethoxazole and an MDR rate of 81.11% in WWTPs [36]. In Japan, ampicillin-resistant *E. coli* showed the highest prevalence among antibiotic-resistant strains, followed by those of levofloxacin, cefotaxime, ceftazidime, and tetracycline in WWTPs [37].

Bacteria carrying extended-spectrum beta-lactamases (ESBL) commonly possess one of the three gene types *bla*_TEM_, *bla*_CTX-M_, and *bla*_SHV_, thereby enhancing their ability to develop resistance against β-lactam antibiotics [38]. Moreover, ESBL-producing *E. coli* are more inclined to exhibit resistance to multiple drugs than *E. coli* that do not produce ESBL [39]. In the present study, the majority of MDR *E. coli* strains possessed *bla*_TEM_ and *bla*_CTX-M_, with the presence of *bla*_SHV_ notably lacking. Within the CTX-M groups, CTX-M-1 and CTX-M-9 emerged as the prevalent types, consistent with trends observed in previous studies conducted in Korea [40]. One significant observation was the predominant detection of CTX-M-8 in MDR *E. coli* strains associated with phylogroup D. Specifically, CTX-M-8 was detected in 100% of the ST38 strains, which are ExPEC lineages. This emphasizes the importance of understanding the genetic factors that determine antibiotic resistance in distinct bacterial lineages.

Correlation analysis between phenotype and genotype showed significant associations between cephalosporins, such as cefoxitin, ceftazidime, and ceftiofur, and the *bla*_TEM_ and *bla*_CMY-2_ genes, and between nalidixic acid and the *qnrS* gene, consistent with previous research findings [41,42]. In the case of streptomycin resistance, it has been found to be closely associated not only with *strA* and *strB* but also with *tetA*, *tetB*, and *bla*_TEM_. Conversely, cases where the phenotypic characteristics of *E. coli* antibiotic resistance did not align with the genotypic features were frequently observed, consistent with previous research [43]. This phenomenon is explained by the increased frequency and sustained presence of MDR isolates, even when antibiotic selection pressure is absent, due to the influence of co-selection mechanisms [43].

Beyond examining antibiotic-resistance mechanisms through phenotypic and molecular analyses, assessing bacterial virulence profiles has emerged as a paramount concern in contemporary microbiological research. Virulence in *E. coli* is orchestrated through mechanisms, such as adhesion, toxin synthesis, the synthesis of polysaccharide capsules, iron acquisition via siderophores, invasion, and the production of additional factors designed to target immune cells [38]. We established that 97.3% of MDR *E. coli* strains harbored *fimH*, whereas 59.9% harbored *ibeA* and *traT.* The potential surface virulence factor *fimH* is common in *E. coli*, and *fimH* mediates cell adhesion, thereby assisting in the formation of bacterial biofilms [44]. Virulence genes exhibited a slightly higher prevalence in isolates from hospital-acquired infections than in isolates from community-acquired infections, with *fimH* (71.8%) and *fyuA* (68.2%) being the most commonly distributed virulence genes [45]. In the present study, MDR *E. coli* displayed a high prevalence of *fimH,* which is associated with cell attachment. However, the ability to form biofilms was mostly absent or weak. Specifically, 69% of MDR *E. coli* formed no biofilm, whereas approximately only 3% formed strong biofilms. This finding contrasts with recent assessments of biofilm formation in fish processing facilities [46], where the majority of organisms showing moderate to strong biofilm-forming capabilities were identified as MDR. Conversely, a recent investigation targeting uropathogenic *E. coli* uncovered an inverse correlation between biofilm formation and antibiotic resistance [47]. This implies a cost of resistance to bacterial cells, suggesting that strains with lower resistance may rely on biofilms to enhance survival, as demonstrated in our study. This may explain why the biofilm formation capability of MDR strains was low in this study.

In a recent review by Kocsis et al. [10], the dissemination of extraintestinal pathogenic high-risk international clones of *E. coli* was extensively discussed, with a particular focus on major clones, such as ST131, ST1193, ST38, ST10, ST69, ST73, ST405, ST410, and ST457. Our study confirmed that MDR bacteria from WWTPs were mainly ExPEC strains, and 44.6% of the isolated *E. coli* strains, which were resistant to six or more antibiotic classes, were linked to the high-risk international clones highlighted by Kocsis et al. Furthermore, as reported by Wang et al. [48], MLST analysis confirmed the predominant association between human-derived *E. coli* strains and ST1193, ST73, ST648, ST131, ST10, and ST1668. Additionally, a Danish study [49] revealed that 38% of ESBL-producing *E. coli* isolates from patients were epidemic MDR *E. coli* ST131 within the B2 group. Consequently, we deduced that the predominant MDR *E. coli* isolated from the WWTPs correlated with human-derived strains. However, this study did not specifically focus on hospital wastewater; instead, it encompassed the entirety of domestic wastewater generated in urban areas. Therefore, there are some limitations to considering this solely due to human-derived pathogenic *E. coli*. Thus, further research spanning from hospital wastewater to the influent of WWTPs is deemed necessary for precise source tracing.

ExPEC strains are generally assigned to phylogroups B2 and D [50]. Moreover, these strains survive in municipal wastewater treatment processes, particularly those associated with ST131 ESBL-producing *E. coli* [51,52]. ST131 and ST1193 within phylogroup B, and ST69 and ST38 within phylogroup D, were predominant in our study. ST131, as documented in studies conducted across Brazil, Nigeria, and Austria, exhibits significant pathogenic potential, MDR, and involvement in infections affecting humans and livestock [53]. ST131 also exhibits a heightened propensity for biofilm formation and the manifestation of antibiotic resistance [54]. ST1193 is a newly recognized worldwide MDR clone with high-risk potential, significantly contributing to community-onset urinary and bloodstream infections. Since 2012, the prevalence of ST1193 has been increasing worldwide, with reports of it replacing ST131 in certain regions [55]. ST38 and several other ExPEC lineages have emerged as a predominant strain in recent years and have been consistently isolated from extraintestinal infections worldwide [56].

Recent findings indicated that ExPECs, including clinically significant strains of urinary pathogenic *E. coli*, are present in treated wastewater effluents and demonstrate a specific adaptation to endure wastewater treatment procedures [51]. Therefore, we may have observed a high proportion of ExPECs among the MDR bacterial strains isolated from WWTPs. MDR ExPECs pose a potential public health risk that necessitates continuous monitoring. Further research is needed to manage antibiotic resistance within WWTPs. Such efforts are expected to play a crucial role in minimizing the impact of antibiotic resistance on public health.

The findings of this study highlighted the critical need for enhanced strategies to combat antibiotic resistance in wastewater treatment plants. The identification of high-risk MDR *E. coli* strains, such as ST131, ST1193, ST38, and ST69, underscores the urgency of implementing advanced treatment technologies and stricter regulatory measures. Currently, research is underway to effectively reduce antibiotic resistance and resistance genes using advanced technologies, such as advanced oxidation processes, membrane filtration, and UV disinfection [57], and such research should continue in a preventive capacity in the future. Moreover, routine monitoring and surveillance programs must be established to track the evolution and dissemination of antibiotic-resistant bacteria and antibiotic-resistant genes within WWPTs. Stakeholders, including policymakers, public health officials, and WWTP operators, can leverage these results to inform targeted inventions and policy frameworks aimed at mitigating the spread of antibiotic resistance. By adopting a multi-faceted approach that combines technological advancements, stringent regulations, and continuous monitoring, it is possible to significantly curb the proliferation of AMR and safeguard public health.

## 5. Conclusions

This study represents the first comprehensive analysis of antibiotic resistance in MDR *E. coli* isolated from WWTPs using culture-based methods. Through examination of antibiotic resistance genes, virulence genes, biofilm formation ability, and *E. coli* typing, we confirmed a close association between MDR *E. coli* from WWTPs and human-derived strains, particularly extraintestinal pathogenic *E. coli*, such as ST131, ST1193, ST38, and ST69. These findings emphasize the crucial importance of managing antibiotic resistance in WWTPs and underscore the necessity for ongoing monitoring and further research to mitigate the public health impact.

## Figures and Tables

**Table 1:** Prevalence of virulence genes in multidrug-resistant *E. coli*.

Virulence Gene	*fimH*	*papC*	*papGII*	*eaeA*	*afa/draBC*	*iroN*	*fyuA*	*east1*	*ibeA*	*traT*	*KpsMTII*
Total (*n* = 74) (%)	72 (97.3%)	5 (6.7%)	1 (1.4%)	5 (6.8%)	8 (10.8%)	11 (14.9%)	44 (59.5%)	10 (13.5%)	3 (4.1%)	44 (59.5%)	32 (43.2%)

**Table 2:** Profiles of extraintestinal pathogenic sequence types from wastewater treatment plants.

ST Type	Strain No.	Biofilm	Antibiotic Resistance	Resistant Genes	Virulence Genes
ST131 (B2)	6–13	weak	AMP-FEP-XNL-CHL-TET-STR-FIS	*sul1*, *sul3*, *tetA*, *aadA*, *CTX-M-9*, *bla_TEM_*, *qnrS*	*iroN*, *ibeA*, *fimH*, *papC*, *fyuA*, *traT*
	12–19	weak	AMP-CIP-NAL-TET-STR-FIS-SXT	*tetA*, *strA*, *strB*, *CTX-M-9*, *bla_TEM_*	*kpsMTII*, *fimH*, *fyuA*, *traT*
	12–82	weak	AMP-CIP-NAL-TET-STR-FIS	*tetA*, *strA*, *strB*, *CTX-M-1*, *CTX-M-9*, *bla_TEM_*	*fimH*, *fyuA*, *traT*
	12–95	weak	AMP-XNL-NAL-TET-STR-FIS-SXT	*tetA*, *strA*, *strB*, *CTX-M-9*, *bla_TEM_*	*kpsMTII*, *fimH*, *afa/draBC*, *fyuA*, *traT*
	14–55	weak	AMP-FEP-CAZ-XNL-CIP-NAL-TET-STR-FIS-SXT	*sul1*, *sul2*, *tetA*, *strA*, *strB*, *CTX-M-1*, *bla_TEM_*	*kpsMTII*, *fimH*, *fyuA*, *traT*
	19–19	non	AMP-FEP-CAZ-XNL-CIP-NAL-TET-STR-FIS	*sul3*, *tetA*, *strA*, *strB*, *CTX-M-1*, *CTX-M-9*, *bla_TEM_*	*kpsMTII*, *fimH*, *afa/draBC*, *fyuA*, *traT*
	24–112	non	AMP-CIP-NAL-TET-STR-FIS-SXT	*sul1*, *sul2*, *tetA*, *strA*, *strB*, *bla_TEM_*	*kpsMTII*, *fimH*, *fyuA*, *traT*
	52–111	non	AMP-FEP-CAZ-XNL-NAL-TET-STR-FIS-SXT	*sul1*, *sul2*, *tetA*, *strA*, *strB*, *bla_TEM_*	*kpsMTII*, *fimH*, *afa/draBC*, *fyuA*, *traT*
ST1193 (B2)	3–4	weak	AMP-CIP-NAL-TET-GEN-STR-FIS-SXT	*sul2*, *tetA*, *strA*, *strB*, *aadA*, *CTX-M-1*, *bla_TEM_*	*kpsMTII*, *fimH*, *fyuA*
	3–5	strong	AMP-CIP-NAL-TET-GEN-STR-FIS-SXT	*sul2*, *tetA*, *strA*, *strB*, *CTX-M-1*, *bla_TEM_*	*kpsMTII*, *fimH*, *fyuA*
	28–8	non	AMP-FEP-CAZ-XNL-CIP-NAL-TET-STR-FIS-SXT	*sul2*, *tetB*, *strA*, *strB*, *aadA*, *CTX-M-1*, *bla_TEM_*	*kpsMTII*, *fimH*, *fyuA*
	32–19	non	AMP-XNL-CIP-NAL-TET-STR-FIS-SXT	*sul1*, *sul2*, *tetA*, *strA*, *strB*, *CTX-M-9*	*kpsMTII*, *fimH*, *fyuA*
	32–29	non	AMP-XNL-CIP-NAL-TET-STR-FIS-SXT	*sul1*, *sul2*, *tetA*, *strA*, *strB*, *CTX-M-9*	*kpsMTII*, *fimH*, *fyuA*
	32–34	non	AMP-XNL-CIP-NAL-TET-STR-FIS-SXT	*sul1*, *sul2*, *tetA*, *strA*, *strB*, *CTX-M-9*	*kpsMTII*, *fimH*, *fyuA*
	61–9	non	AMP-FEP-CAZ-XNL-CIP-NAL-TET-GEN-STR-FIS-SXT	*sul1*, *sul2*, *tetA*, *strA*, *strB*, *CTX-M-9*, *bla_TEM_*_,_*qnrS*	*kpsMTII*, *fimH*, *fyuA*
	64–78	non	AMP-XNL-CIP-NAL-TET-GEN-STR-FIS-SXT	*sul1*, *sul2*, *tetA*, *strA*, *strB*, *CTX-M-1*, *bla_TEM_*	*kpsMTII*, *fimH*, *fyuA*
ST69 (D)	19–144	non	AMC-AMP-FOX-CAZ-XNL-NAL-TET-GEN-FIS-SXT	*tetB*, *CTX-M-1*	*kpsMTII*, *fimH*, *fyuA*, *traT*
	29–6	non	AMC-AMP-FOX-CAZ-XNL-NAL-GEN-STR-FIS-SXT	*sul1*, *CTX-M-1*, *CTX-M-9*, *CMY-2*	*fimH*, *fyuA*, *traT*
	44–171	non	AMP-CIP-NAL-TET-STR-FIS-SXT	*sul2*, *tetB*, *strA*, *strB*	*fimH*, *fyuA*, *traT*
ST38 (D)	6–86	non	AMC-AMP-FEP-XNL-CIP-NAL-TET-GEN-FIS-SXT	*tetB*, *CTX-M-8*, *CTX-M-9*, *bla_TEM_*	*kpsMTII*, *fimH*, *fyuA*, *traT*
	11–144	non	AMP-FEP-XNL-CIP-NAL-GEN-FIS-SXT	*sul2*, *CTX-M-2*, *CTX-M-8*, *CTX-M-9*, *bla_TEM_*	*kpsMTII*, *fimH*, *fyuA*
	12–117	moderate	AMP-FEP-XNL-CIP-NAL-GEN-FIS-SXT	*sul1*, *CTX-M-8*, *CTX-M-9*	*kpsMTII*, *fimH*, *fyuA*
	13–69	non	AMP-NAL-TET-STR-FIS-SXT-COL	*sul1*, *sul2*, *tetA*, *strA*, *strB*, *CTX-M-8*, *bla_TEM_*	*kpsMTII*, *fimH*, *afa/draBC*, *fyuA*, *traT*
	17–40	weak	AMC-AMP-FEP-FOX-XNL-CHL-CIP-NAL-TET-GEN-STR-FIS-SXT	*sul1*, *sul2*, *strA*, *strB*, *CTX-M-8*, *CTX-M-9*, *bla_TEM_*, *CMY-2*	*kpsMTII*, *fimH*, *afa/draBC*, *fyuA*, *traT*, *east1*
	17–79	weak	AMP-CIP-NAL-TET-STR-FIS-SXT-COL	*tetA*, *strA*, *strB*, *CTX-M-8*, *CTX-M-9*, *bla_TEM_*	*kpsMTII*, *fimH*, *fyuA*, *traT*
	54–4	non	AMP-XNL-CIP-NAL-STR-FIS	*sul2*, *strA*, *strB*, *CTX-M-8*, *CTX-M-9*, *qnrS*	*kpsMTII*, *fimH*, *fyuA*
	61–113	non	AMP-XNL-CIP-NAL-STR-FIS-SXT	*sul1*, *sul2*, *strA*, *strB*, *CTX-M-8*, *CTX-M-9*	*kpsMTII*, *fimH*, *fyuA*

**Table 3:** Residual antibiotic concentrations in the effluent samples from wastewater treatment plants during dry and wet seasons.

Antibiotic Agents	The Number of Detection Points among 33 WWTPs (Unit)		Average Concentration (µg/L)		Median Concentration (µg/L)	
	Dry Season	Wet Season	Dry Season	Wet Season	Dry Season	Wet Season
Ciprofloxacin	33	33	0.120	0.056	0.102	0.048
Sulfamethoxazole	33	33	0.070	0.103	0.066	0.085
Trimethoprim	32	33	0.050	0.026	0.044	0.015
Ceftazidime	16	15	0.111	0.209	0.068	0.162
Tetracycline	10	5	0.108	0.054	0.095	0.052
Cefepime	2	6	0.188	0.206	0.188	0.139
Meropenem	2	-	0.556	-	0.556	-

## Data Availability

The original contributions presented in the study are included in the article/Supplementary Materials, further inquiries can be directed to the corresponding author/s.

## Supplementary Materials

The following supporting information can be downloaded at: https://www.mdpi.com/article/10.3390/microorganisms12061119/s1. Table S1: List of primers and PCR conditions used in this study. Table S2: Comprehensive analysis data of *Escherichia coli* resistant to six or more antibiotic classes (*n* = 74).
