# Supplementary material for: Comprehensive Assessment of Multidrug-Resistant and Extraintestinal Pathogenic Escherichia coli in Wastewater Treatment Plant Effluents
Source: Microorganisms. 2024 May 31;12(6):1119. doi: 10.3390/microorganisms12061119 (PMC11205404; doi:10.3390/microorganisms12061119)
Supplement: Supplementary file 1 [file microorganisms-12-01119-s001.zip › Supplementary Material_Table S1.pdf]

Table S1 – List of primers and PCR conditions used in this study.

	Target	Primer Name	Primer Sequence	Size (pb)	*Annealing* (T)	Ref.
tetracycline	*tetA*	tetA-F	5’-GCTACATCCTGCTTGCCTTC–3’	210	55°C	[17]
		tetA-R	5’-CATAGATCGCCGTGAAGAGG–3’			
	tetB	tetB-F	5’-TTGGTTAGGGGCAAGTTTTG–3’	659	55°C	[17]
		tetB-R	5’-GTAATGGGCCAATAACACCG–3’			
aminoglycoside	*strA*	strA-F	5’-CCTGGTGATAACGGCAATTC–3’	546	55°C	[18]
		strA-R	5’-CCAATCGCAGATAGAAGGC–3’			
	strB	strB-F	5’-ATCGTCAAGGGATTGAAACC–3’	509	55°C	[18]
		strB-R	5’-GGATCGTAGAACATATTGGC–3’			
	aadA	aadA1-F	5’-TTTGCTGGTTACGGTGAC–3’	497	68°C	[19]
		aadA2-R	5’-GCTCCATTGCCCAGTCG–3’			
sulfonamides	*sul1*	Sul1-L	5’-GTGACGGTGTTCGGCATTCT–3’	413	55°C	[20]
		Sul1-R	5’-TCCGAGAAGGTGATTGCGCT–3’			
	sul2	Sul2-L	5’-CGGCATCGTCAACATAACCT–3’	657	56°C	[20]
		Sul2-R	5’-TGTGCGGATGAAGTCAGCTC–3’			
	sul3	Sul3-F	5’-GAGCAAGATTTTTGGAATCG–3’	412	55°C	[20]
		Sul3-R	5’-CATCTGCAGCTAACCTAGGGCTTTGGA–3’			
beta-lactamase	*TEM*	TEM-F	5’-GCGGAACCCCTATTTG–3’	964	55°C	[22]
		TEM-R	5’-ACCATTGCTTAATCAGTGAG–3’			
	OXA-1	OXA-1-F	5’-ATGAAAAACACAATACATATCAACTTCGC–3’	820	55°C	[22]
		OXA-1-R	5’-GTGTGTTTAGAATGGTGATCGCATT–3’			
	OXA-2	OXA-2-F	5’-ACGATAGTTGTGGCAGACGAAC–3’	601	55°C	[22]
		OXA-2-R	5’-ATYCTGTTTGGCGTATCRATATTC–3’			
	SHV	SHV-F	5’-AGGATTGACTGCCTTTTTG–3’	393	55°C	[23]
		SHV-R	5’-ATTTGCTGATTTCGCTCG–3’			
	CMY-2	CMY-F	5’-GACAGCCTCTTTCTCCACA–3’	1000	55°C	[23]
		CMY-R	5’-TGGACACGAAGGCTACGTA–3’			
	CTX-M-1	CTX-M-1F	5’-CAGAGATTTTGCCGTCTAAG–3’	945	55°C	[21]
		CTX-M-1R	5’-GGCCCATGGTTAAAAAATCACTGC–3’			
	CTX-M-2	CTX-M-2F	5’-CTCAGAGCATTCGCCGCTCA–3’	843	55°C	[21]
		CTX-M-2R	5’-CCGCCGCAGCCAGAATATCC–3’			
	CTX-M-8	CTX-M-8F	5'-ACTTCAGCCACACGGATTCA-3'	1024	55°C	[21]
		CTX-M-8R	5'-CGAGTACGTCACGACGACTT-3'			
	CTX-M-9	CTX-M-9F	5’-GTTACAGCCCTTCGGCGATGATTC–3’	876	55°C	[21]
		CTX-M-9R	5’-GCGCATGGTGACAAAGAGAGTGCAA–3’			
	CTX-M-25	CTX-M-25F	5'-GCACGATGACATTCGGG-3'	327	55°C	[21]
		CTX-M-25R	5'-AACCCACGATGTGGGTAGC-3'			
quinolone	*qnrA*	QnrA-F	5’-AGAGGATTTCTCACGCCAGG-3	580	58°C	[24]
		QnrA-r	5’-TGCCAGGCACAGATCTTGAC-3			
	qnrB	QnrB-F	5’-GGMATHGAAATTCGCCACTG-3’	264	58°C	[24]
		QnrB-r	5’-TTTGCYGYYCGCCAGTCGAA-3’			
	qnrC	QnrC-F	5’-GCGAATTTCCAAGGGGCAAA-3’	135	58°C	[24]
		QnrC-r	5’-ACCCGTAATGTAAGCAGAGCAA-3’			
	qnrD	QnrD-F	5′-AGGTGTAGCATGTATGGAAAAGC-3’	691	58°C	[24]
		QnrD-r	5′-ACATTGGGGCATTAGGCGTT-3’			

	qnrS	QnrS-F	5'-GCAAGTTCATTGAACAGGGT-3'	428	58°C	[24]
		QnrS-r	5'-TCTAAACCGTCGAGTTCGGCG-3'			
	qnrVC	QnrVC-F	5'-GAGYTKTATGGTTTAGAYCCTCG-3'	71	58°C	[24]
		QnrVC-r	5'-TGTTCYTGYTGCCACGARCA-3'			
Aminoglycoside	*fimH*	fimH-F	5'-TGCAGAACGGATAAGCCGTGG-3'	508	63°C	[27]
		fimH-R	5'-GCAGTCACCTGCCCTCCGGTA-3'			
	papC	papC-F	5'-GTGGCAGTATGAGTAATGACCGTTA-3'	205	63°C	[27]
		papC-R	5'-ATATCCTTTCTGCAGGGATGCAATA-3'			
	papGII	papGII-F	5'-GGGATGAGCGGGCCTTTGAT-3'	190	63°C	[27]
		papGII-R	5'-CGGGCCCCCAAGTAACTCG-3'			
	eaeA	eaeA-F	5'-GACCCGGCACAAGCATAAGC-3'	384	55°C	[25]
		eaeA-R	5'-CCACCTGCAGCAACAAGAGG-3'			
	afa/draBC	afa/draBC-F	5'-GGCAGAGGGCCGGCAACAGGC-3'	559	63°C	[27]
		afa/draBC-R	5'-CCCGTAACGCGCCAGCATCTC-3'			
	iroN	iroN-F	5'-AAGTCAAAGCAGGGGTTGCCCG-3'	665	63°C	[27]
		iroN-R	5'-GACGCCGACATTAAGACGCAG-3'			
	fyuA	fyuA-F	5'-TGATTAACCCCGCGACGGGAA-3'	880	63°C	[26]
		fyuA-R	5'-CGCAGTAGGCACGATGTTGTA-3'			
	stx1	stx1-F	5'-ACACTGGATGATCTCAGTGG-3'	614	55°C	[25]
		stx1-R	5'-CTGAATCCCCCTCCATTATG-3'			
	stx2	stx2-F	5'-CCATGACAACGGACAGCAGTT-3'	779	55°C	[25]
		stx2-R	5'-CCTGTCAACTGAGCACTTTG-3'			
	east1	east 11a	5'-CCATCAACACAGTATATCCGA-3'	111	45°C	[26]
		east 11b	5'-GGTCGCGAGTGACGGCTTTGT-3'			
	ibeA	ibeA-F	5'-AGGCAGGTGTGCGCCGCGTAC-3'	171	63°C	[27]
		ibeA-R	5'-TGGTGCTCCGGCAAACCATGC-3'			
	ipaH	ipaHIII	5'-GTTCCTTGACCGCCTTTCCGATACCGTC-3'	600	50°C	[26]
		ipaHIV	5'-GCCGGTCAGCCACCCTCTGAGATAC-3'			
	traT	TraT-F	5'-GGTGTGGTGCGATGAGCACAG-3'	290	63°C	[26]
		TraT-R	5'-CACGGTTCAGCCATCCCTGAG-3'			
	kpsMT II	kpsMT II-F	5'-GCGCATTTGCTGATACTGTTG-3'	272	63°C	[27]
		kpsMT II-R	5'-CATCCAGACGATAAGCATGAGCA-3'			
